# Evaluation of First-Week Fluid Intake and Maximal Weight Loss Percentage with In-Hospital Adverse Outcomes Among Moderately and Very Preterm Newborns in Ethiopia

**DOI:** 10.3390/children12070872

**Published:** 2025-07-01

**Authors:** Gregory C. Valentine, Tessa Rue, Olivia C. Brandon, Krystle M. Perez, Thomas R. Wood, Sharla Rent, Gal Barbut, Merhawit Abadi, Redeat Workneh, Gesit Metaferia, Mahlet Abayneh

**Affiliations:** 1 Division of Neonatology, University of Washington, Seattle Children’s Hospital, Seattle, WA 98195, USA; ruet@uw.edu (T.R.); obrandon@uw.edu (O.C.B.); kmperez@uw.edu (K.M.P.); tommyrw@uw.edu (T.R.W.); 2 Division of Neonatology, Duke University, Durham, NC 27708, USA; sharla.rent@duke.edu; 3 Division of Neonatology, University of Rochester, Rochester, NY 14627, USA; gal_barbut@urmc.rochester.edu; 4 Division of Neonatology, St. Paul’s Hospital Millennium Medical College, Addis Ababa 1165, Ethiopia; merhawit.abadi@sphmmc.edu.et (M.A.); redeat.workneh@sphmmc.edu.et (R.W.); gesit.metaferia@sphmmc.edu.et (G.M.); mahlet.abayneh@sphmmc.edu.et (M.A.)

**Keywords:** weight loss, intravenous fluids, weight change, neonatology, prematurity, dehydration, Ethiopia

## Abstract

**Background/Objective:** We sought to address ongoing gaps in understanding the relationship between first-week percent maximal weight loss (MWL) and average first-week total fluid intake (TFI), enteral intake, and parenteral intake among premature newborns with adverse in-hospital outcomes born in low- and middle-income countries (LMICs). **Methods:** We evaluated newborns born <34 weeks gestation or <1500 g who survived at least 7 days at the St. Paul’s Hospital Millennium Medical College (SPHMMC) neonatal intensive care unit in Ethiopia. We performed univariate and multivariate regression models analyzing the first-week MWL, average TFI, parenteral, and enteral intake and their relationships with adverse in-hospital outcomes. **Results:** Among N = 490 moderately and very preterm newborns, multivariate regression models demonstrated that >13% MWL was associated with significantly increased odds of suspected necrotizing enterocolitis (NEC), culture-positive sepsis, retinopathy of prematurity (ROP), and a longer length of stay (LOS). An average *enteral* intake of >60 mL/kg/day was significantly associated with reduced odds of all-cause mortality, suspected NEC, culture-positive sepsis, ROP, and a shorter LOS, whereas an average *parenteral* intake of >60 mL/kg/day was associated with increased odds of in-hospital mortality, culture-positive sepsis, ROP, and a longer LOS. **Conclusions:** In moderately and very preterm neonates in an LMIC setting, >13% MWL is associated with adverse health outcomes. Increasing the average parenteral intake over the first week after birth among moderately and very preterm neonates is significantly associated with adverse in-hospital outcomes whereas increasing the average enteral intake is associated with improved outcomes.

## 1. Introduction 

The majority of preterm births occur in low- and middle-income countries, with the highest incidence occurring in sub-Saharan Africa [1]. Among premature newborns, the first week after birth represents a critical and vulnerable fetal-to-neonatal transitional period and coincides with the time period when the majority of neonatal deaths occur [2,3]. The premature newborn is at high risk for dehydration and malnutrition given high insensible fluid losses due to their immature skin function, increased respiratory rate, immature kidney function, and limited enteral feeding, which are all especially prominent during the fetal-to-neonatal transition period [4]. Thus, the provision of adequate nutritional-fluid support via enteral feeds and parenteral fluids is of critical importance to the care of moderately and very premature newborns during this transitional period. Yet, there exists a paucity of published literature on the optimal enteral and parenteral fluid support for premature newborns in sub-Saharan Africa, leading to a wide variation of feeding practices across countries and contexts [5]. Some common feeding practices for vulnerable preterm neonates across countries such as Nigeria, Kenya, and Ethiopia include the initiation of small volume enteral feeds (as available) along with parenteral fluids with subsequent progressive advancement of enteral feeds until parenteral fluids can be discontinued [6].

Early human-milk enteral feeding and improved nutritional support of premature newborns in the first week after birth have been strongly associated with improved short- and long-term outcomes [7,8,9,10,11,12,13]. Yet, increasing the first-week average total fluid intake (TFI) and an altered fluid balance is associated with adverse outcomes including all-cause mortality, bronchopulmonary dysplasia (BPD), necrotizing enterocolitis (NEC), and patent ductus arteriosus (PDA), requiring surgical intervention among extremely preterm newborns in the United States [9,10,14,15]. Noting that TFI includes both enteral feeds and parenteral fluids, there remains a paucity of evidence detailing the relative contributions of enteral feeding versus parenteral fluids on outcomes among premature newborns [16,17]. Furthermore, evidence has largely been derived in high-resource contexts without generalizability to low- and middle-income countries (LMICs) where resources to mitigate severe fluid shifts may not exist [4]. Thus, there is a critical gap in evidence related to assessing the contributions of TFI, parenteral fluid and enteral feeding components, and maximal weight loss (MWL) percentage on in-hospital outcomes of moderately and very preterm newborns in LMICs such as Ethiopia. 

We established a neonatal nutrition and outcomes database at St. Paul’s Hospital Millennium Medical College (SPHMMC) in Ethiopia to address these needs. We performed a cross-sectional, observational study to evaluate the association between the first-week average TFI, parenteral fluid intake, enteral feeding intake, and MWL percentage with adverse in-hospital neonatal outcomes among moderately and very preterm newborns, defined as <34 weeks gestation and/or <1500 g birthweight. We hypothesized that increasing the average TFI, increasing the average parenteral fluid intake, and reducing the average enteral intake over the first 7 days after birth and an excessive MWL are all associated with adverse in-hospital outcomes among moderately and very preterm newborns in Ethiopia. 

## 2. Subjects and Methods

### 2.1. Overview of Database and Quality Control Assessments 

A nutrition and outcomes database was developed using the Research Electronic Data Capture (REDCap) online database through the University of Washington (UW) and implemented at SPHMMC neonatal intensive care unit in Addis Ababa, Ethiopia in 2022. A dedicated data assistant was hired as part of this project. SPHMMC is the second largest hospital in Ethiopia and serves as a tertiary referral hospital throughout the Ethiopian capital, serving approximately 5 million individuals. SPHMMC supports >12,000 deliveries annually, and the SPHMMC 60-bed NICU is staffed by 2 neonatologists, 4 neonatal-perinatal medicine fellows, and additional resident physicians, clinical officers, medical students, nurses, nurse educators and ancillary staff. The database included over 1080 variables per patient, including demographic and clinical variables per patient. Local feeding and fluid guidelines are seen in Supplemental Tables S1 and S2. 

The quality control process included training on proper extraction of data variables from paper charts with random audits. Daily audits of data entry were performed for the first month of the database implementation with subsequent biweekly and then monthly audits including at least 5% of randomly selected charts. 

Patient data was entered into the REDCap database at the time of death, transfer, discharge, or family decision to leave against medical advice. A waiver of informed consent was obtained and approved by the SPHMMC and UW Institutional Review Boards. 

### 2.2. Time Period, Inclusion and Exclusion Criteria

Data analyzed include neonates born between February 2022 and July 2024. The analyses were restricted to only the babies who met the following inclusion criteria: (a) birthweight <1500 g or were a moderately or very preterm neonate (defined as <34 weeks gestational age by best obstetric estimate), (b) length of hospitalization ≥7 days, (c) had ≥3 daily weight measurements within the first 7 days after birth, and (d) arrived in the SPHMMC NICU within the first day after birth. 

### 2.3. Defining Exposures and Outcomes

We calculated MWL as the lowest measured daily weight in the first 7 days after birth as a percentage of birthweight measured in the NICU. Babies with no weight loss or those that had weight gain (n = 22) were reported as having a MWL of zero. Newborns were considered to have regained birthweight if any weight after the day of MWL was at least equal to birthweight. Percent change from birthweight was calculated for each daily weight and LOESS (Locally Estimated Scatterplot Smoothing)-smoothed 10th, 50th, and 90th percentiles were obtained at each day after birth across the sample, stratified by gestational age.

We also assessed average fluids administered per kg birthweight in the first seven days after birth. Total daily fluids were entered by hospital and research staff. Daily enteral feeds were calculated separately as feeding amount times frequency of feeds. Daily parenteral fluids include all intravenous (IV) boluses, IV medications, and all blood products. Of note, SPHMMC does not have TPN, and IV fluids are generally dextrose-containing. Peripheral IVs are used to provide infusions.

Outcomes were defined as the following: (a) all-cause mortality included any case of mortality occurring during hospitalization, (b) suspected NEC was based on clinical judgment utilizing X-rays to evaluate for pneumatosis intestinalis and clinical findings such as blood in the stool, (c) pulmonary hemorrhage was defined clinically, with blood in the endotracheal tube or observed in the oropharynx, (d) culture-positive sepsis was a positive blood culture that was consistent with a true infection, (e) retinopathy of prematurity (ROP) was defined by a clinical ophthalmologist and included any stage ROP, and (f) length of stay (LOS) was the date of death, transfer, or discharge minus the date of admission and expressed in days. For LOS, newborns that died were imputed as a LOS of 60 days which corresponded with the longest hospital duration for any survivor.

### 2.4. Statistical Analyses

**Figure 1:**
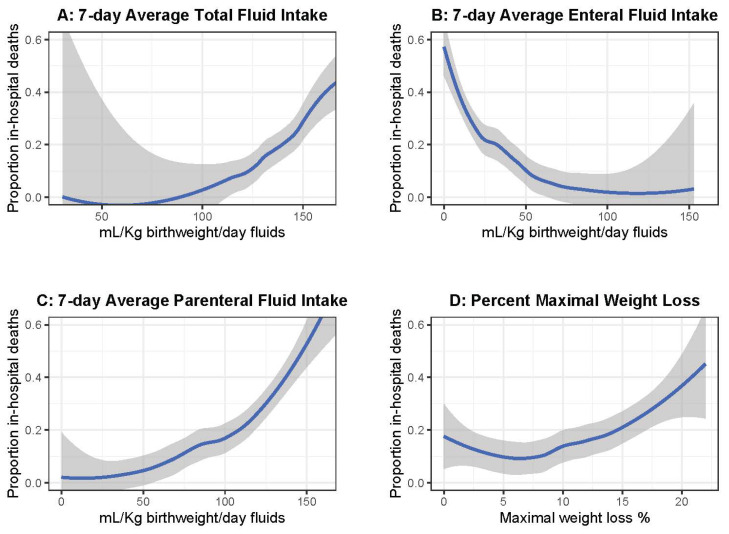
Proportion of in-hospital deaths after first week by (**A**) 7-day average total fluid intake, (**B**) 7-day average enteral feeding intake, (**C**) 7-day average parenteral fluid intake, and (**D**) percent maximal weight loss (MWL) in the first 7 days after birth. Gray band represents the 95% CI around the LOESS-smoothed curve. From these curves, we obtained categorical cut-offs of (**A**) >110 mL/kg birthweight/day of TFI vs. ≤110 mL/kg birthweight/day, (**B**) >60 mL/kg birthweight/day of enteral feeding intake vs. ≤60 mL/kg birthweight day, (**C**) >60 mL/kg birthweight/day of parenteral fluid intake vs. ≤60 mL/kg birthweight day, and (**D**) >13% MWL vs. ≤13% MWL for subsequent categorical evaluation of these exposures with in-hospital outcomes.

By inclusion, all infants survived the first week after birth. LOESS-smoothed estimates were obtained for the proportion of in-hospital deaths after the first week with 95% confidence intervals by MWL, 7-day average TFI, 7-day average parenteral fluids, and 7-day average enteral feeds (Figure 1). These curves informed the choice of cut-offs for categorization of MWL and fluids administered for analysis of hospital outcomes as categorical analyses. 

Pregnancy and birth characteristics were compared between MWL categories. Characteristics nominally associated with MWL (*p* < 0.05) were included in all models of hospital outcomes to adjust for differences. Of note, while intrauterine growth restriction was significantly associated with MWL, it was not included as a covariate due to collinearity with birthweight and gestational age, which were already included in the multivariate regression models. Additionally, we included respiratory support need during hospitalization and 5-min Apgar score as additional covariates for further inclusion of severity of illness metrics.

To assess possible associations between MWL category and hospital outcomes, we used logistic regression models for binary hospital outcomes (mortality, suspected NEC, culture-positive sepsis, ROP, and pulmonary hemorrhage) and linear regression models for log-transformed length of stay. All regression models included MWL categories, total fluids administered in the first seven days after birth, gestational age, birthweight, and indicators for multiple births, c-sections, and pre-eclampsia. We also performed a sensitivity analysis evaluating MWL of <5% given studies reporting that limited-to-no weight loss after birth is associated with adverse outcomes [10,14].

The same approach was used to assess associations between fluid categories and hospital outcomes except MWL replaced total fluids administered as an adjustment variable. For models of enteral/parenteral fluid categories, additional adjustment was made for parenteral/enteral fluids using residuals from a regression on enteral/parenteral. R was used for data analysis (R Foundation for Statistical Computing, Vienna, Austria) [18]. A probability of less than 0.05 was considered significant. 

## 3. Results 

### 3.1. Determining Categories of MWL, TFI, Parenteral and Enteral Intake for Outcome Assessment

A total of N = 490 moderately or very preterm newborns met the inclusion criteria for analyses, including neonates as low as 25 weeks gestation. The flow diagram of those meeting inclusion and exclusion criteria is seen in Supplemental Figure S1 along with a sensitivity analysis evaluating the maternal and infant factors between those included versus excluded (Supplemental Table S3). We first performed LOESS smooth plots with 95% confidence intervals for MWL, 7-day average TFI, 7-day average parenteral intake, and 7-day average enteral intake to determine the inflection points of difference and where the proportion of mortality did not include 0 (Figure 1). The categorical cut-offs were as follows: MWL >13% vs. ≤13%, 7-day average TFI >110 mL/kg birthweight/day vs. ≤110 mL/kg birthweight/day, 7-day average parenteral intake >60 mL/kg birthweight/day vs. ≤60 mL/kg birthweight/day, and 7-day average enteral intake >60 mL/kg birthweight/day vs. ≤60 mL/kg birthweight/day. The characteristics of maternal and neonatal outcomes for those with an MWL >13% or ≤13% are presented in Table 1. 

### 3.2. MWL Differs Across Gestational Age Categories

The percent MWL increased with decreasing gestational age (Supplemental Table S4). Neonates at <30 weeks gestation had the highest median percent MWL at a 14.7% loss; neonates that were 34 weeks but <1500 g had a median percent MWL of 5.4%. The trends in MWL per gestational age category with their 95% confidence intervals depicting the variation are depicted in Supplemental Figure S2. Of note, 22 neonates did not lose any weight from their birthweight. 

### 3.3. Association of Percent MWL and Adverse In-Hospital Outcomes

When evaluating outcomes by percent MWL, newborns with an MWL >13% had significantly increased odds of suspected NEC, culture-positive sepsis, ROP, and a longer length of stay after adjusting for potential confounders compared to newborns with an MWL ≤13% (Table 2). The percent MWL did not reach statistical significance related to all-cause in-hospital mortality or pulmonary hemorrhage. In the sensitivity analysis, which included MWL in three categories (<5%, 5-≤13%, and >13%), similar findings occurred with increased odds of suspected NEC, ROP, and a longer length of stay in those with >13% MWL (Supplemental Table S5).

### 3.4. Association of Total Fluid Intake and Adverse In-Hospital Outcomes

A 7-day average TFI of >110 mL/kg birthweight/day was significantly associated with increased odds of ROP and a longer length of stay, adjusting for potential confounders, and when compared to those with an average TFI of ≤110 mL/kg birthweight/day (Table 3). All cases of pulmonary hemorrhage occurred in those with a TFI of >110 mL/kg birthweight/day. However, TFI was not significantly associated with in-hospital mortality, suspected NEC, or culture-positive sepsis. 

### 3.5. Association of Parenteral and Enteral Intake and Adverse In-Hospital Outcomes

A 7-day average enteral feeding intake of >60 mL/kg birthweight/day was significantly associated with *reduced* odds of all-cause in-hospital mortality, suspected NEC, culture-positive sepsis, ROP, and a shorter length of hospitalization when compared to a 7-day enteral feeding intake of ≤60 mL/kg birthweight/day and adjusting for potential confounders (Table 4). All cases of pulmonary hemorrhage occurred in those with an average enteral feeding intake of ≤60 mL/kg birthweight/day.

A 7-day parenteral fluid intake of >60 mL/kg birthweight/day was significantly associated with *increased* odds of all-cause in-hospital mortality, culture-positive sepsis, ROP, and a longer length of hospitalization; see Table 4. All cases of pulmonary hemorrhage occurred in those with an average parenteral intake of >60 mL/kg birthweight/day. Parenteral fluid intake did not reach statistical significance for suspected NEC, although it approached significance. 

Forest plots for MWL, average TFI, average parenteral intake, and average enteral intake on adverse outcomes are seen in Supplemental Figure S3. 

## 4. Discussion

Among moderately and very preterm newborns in Ethiopia, having >13% MWL from birthweight, >110 mL/kg birthweight/day 7-day average TFI, >60 mL/kg birthweight/day average *parenteral* intake, and ≤60 mL/kg birthweight/day average *enteral* intake are all strongly associated with increased odds of developing adverse health outcomes including mortality, suspected NEC, culture-positive sepsis, ROP, and longer length of hospitalization. The relationship between early nutritional practices with length of stay and associated adverse comorbidities further strains already burdened healthcare systems in resource-limited settings [19]. Our findings underscore the importance of future clinical trials exploring optimal early enteral feeding and nutritional practices for MP neonates in LMICs. 

Our study addressed a significant deficit in existing literature exploring the relative contributions of enteral feeds, parenteral fluids, TFI, and percent MWL on the outcomes of moderately and very preterm newborns in low-resource settings. As >60% of all preterm births and 98% of all neonatal deaths occur in LMICs (with prematurity being the leading cause of death and disability among children under five worldwide [20,21,22,23]), research evaluating simple and accessible strategies to improve short- and long-term outcomes of preterm newborns in LMICs is critical. While our study cannot ascertain causality, our study’s findings highlight the need for future clinical trials to evaluate the role of early feeding and fluid management and their relationship with in-hospital outcomes. It is also possible that upstream conditions such as intestinal intolerance may lead to increased MWL and adverse health outcomes, further underscoring the need for clinical trials to confirm our findings. These future studies should ascertain the relative contributions of enteral feeding and parenteral fluids and the role of early weight loss on neonatal outcomes—with our study being suggestive of a potential strategy that both optimizes enteral feedings while avoiding dramatic early weight loss. Findings from such studies could pave the way to overcoming pervasive health disparities and inequities related to premature newborns in LMICs. 

### 4.1. Relative Contributions of Enteral and Parenteral Intake on Outcomes

While increasing the average TFI in the first week after birth has been consistently associated with adverse in-hospital outcomes among premature newborns in high-resource settings [4,9,10,17,24,25,26], the relative contributions of enteral feeds and parenteral intake on adverse outcomes is a critical gap in the medical literature from LMICs. Our findings suggest that the main driver for the adverse outcomes related to increasing the average first-week TFI is parenteral fluid intake rather than enteral feeds. Additionally, within the multivariate regression models evaluating the average first-week TFI among moderately and very preterm neonates at SPHMMC, only 1 case of mortality (3%) occurred in those with an average TFI of ≤110 mL/kg birthweight/day compared with 82 cases (22%) in those with an average TFI of >110 mL/kg birthweight/day. However, this finding did not reach statistical significance (*p* = 0.21), possibly due to the limited sample size affecting the power. 

To rigorously evaluate the specific relative contributions of enteral and parenteral intake on outcomes, we employed robust statistical methods by including adjustments for the MWL percentage as well as the residuals of the opposite corresponding variable. By doing so, we isolate the independent effects of enteral feeding and parenteral fluid intake on neonatal outcomes by removing their shared variation. Thus, the findings of an increased average first-week parenteral fluid intake being strongly associated with 5-fold increased odds of mortality and increasing the average first-week enteral feeding intake reducing the odds of mortality by 70% are particularly noteworthy. 

These findings are consistent with high-level evidence including systematic reviews and meta-analyses demonstrating that early enteral feeding and limiting enteral fasting improves the well-being of vulnerable, preterm newborns [11,16,27,28]. Several studies and clinical trials have been conducted in India and the US evaluating early total enteral feeding (ETEF), defined as providing *only* enteral feeds as compared to conventional enteral feeding, defined as a mixture of parenteral fluids and enteral feeds, and demonstrating that the risk of overall mortality, NEC, and late-onset sepsis is likely reduced, and shorter lengths of hospitalization are likely among those receiving ETEF [7,29]. However, given the lack of large scale clinical trials, systematic reviews and meta-analyses recommend additional trials to evaluate whether these findings are reproducible and generalizable across differing contexts [30]. 

Furthermore, as parenteral fluids require intravenous (IV) access, and IV access is a leading cause of late-onset sepsis [31,32,33,34], limiting parenteral fluid duration may improve short- and long-term outcomes for the moderately and very preterm neonatal population. In fact, the leading cause of late-onset, culture-positive sepsis among very preterm newborns is skin flora, specifically *Staphylococcus aureus* and coagulase-negative staphylococcus, which make up >50% of all cases of late-onset sepsis [35]. Newborns diagnosed with late-onset sepsis have higher risk of mortality, requiring oxygen therapy, and requiring surgical intervention [35]. Unsurprisingly, prolonged parenteral nutrition and limited enteral nutrition has been reported as risk factors for late-onset sepsis [36]. Thus, our study’s findings add to this expanding body of literature highlighting that reducing parenteral fluid exposure while maximizing enteral feeding in the first week is strongly associated with improving both short- and long-term health and well-being for vulnerable small and/or sick newborns in LMICs. 

### 4.2. Percent MWL and Associations with Adverse Health Outcomes 

Studies in the United States have determined that a percent MWL >15% from birthweight is associated with increased odds of developing adverse outcomes such as NEC [10]. Similar to high-income settings, a MWL >13% from birthweight was significantly associated with nearly 2- to 3-fold increased odds of suspected NEC, culture-positive sepsis, and any diagnosis of ROP, as well as a longer LOS overall in our Ethiopian MP neonatal population. Thus, an MWL beyond 13% from birthweight does appear to be pathologic among MP newborns and should likely be avoided. However, as this study is retrospective, it is also quite possible that this association could simply be due to upstream conditions that could alter the absorption of fluids and nutrients via feeding, thereby leading to a higher MWL as a symptom of a larger issue associated with adverse health outcomes.

Other studies in the United States have reported that MWL has a non-linear relationship with adverse outcomes [10,14,37]. Specifically, premature neonates with minimal-to-no weight loss, i.e., <5%, have similarly increased odds of adverse health outcomes like those with severe weight loss, i.e., >15% [10,14,37]. Our sensitivity analysis suggests a similar trend in this Ethiopian cohort. For example, moderately and very preterm neonates who had an MWL <5 had an estimated 2-fold increased odds of in-hospital mortality compared to an MWL 5-≤13% (Supplemental Table S3), although this did not reach statistical significance (*p* = 0.08). Whether MWL causes adverse health outcomes is unknown. It may be a symptom of another issue such as increased insensible fluid losses, respiratory distress, or intestinal malabsorption, to name a few. Therefore, future clinical trials are of critical importance to ascertain whether MWL is a symptom or a cause of adverse in-hospital outcomes among vulnerable preterm newborns. 

### 4.3. Weight Loss Patterns of Moderately and Very Preterm Newborns at SPHMMC Differ from Those in High-Resourced Settings

Another notable finding is that the overall weight loss patterns in the first week appear specific to the gestational age at birth with a lower gestational age associated with an increasing percent MWL (Supplemental Figure S1). However, we have previously reported that among extremely preterm newborns across 30 NICUs in the United States, there were no substantial differences in weight loss trajectories across newborns born at 24 weeks through 27 weeks gestational age [10]. The difference in findings from the US compared to SPHMMC are likely related to contextual differences such as the lack of humidified incubators, which can increase insensible fluid losses such as transepidermal water loss. As an increasing degree of prematurity is directly associated with an increased degree of insensible fluid losses [38,39], these contextual differences likely contribute to these findings [4] and underscore the importance of evaluating feeding and fluid practices in LMICs as findings from high-income countries may not be generalizable given the contextual differences.

### 4.4. Strengths and Limitations

Our study has several notable strengths, including a large sample size of nearly 500 moderately and very preterm newborns in an LMIC setting, allowing us to detect statistically significant and clinically meaningful differences in outcomes. We also employed a full-time data collector to ensure all data was collected in a robust manner without adding an extra burden on the clinical staff. Additionally, our study rigorously evaluates the relative contributions of enteral feeds and parenteral fluids in the first week and their relationship with outcomes among moderately and very preterm newborns using robust statistical methods. Additionally, we evaluated common maternal and infant risk factors that can affect the fluid and weight loss of neonates, including the mode of delivery, antenatal corticosteroids, growth restriction, and other factors. These were evaluated and included in the statistical models if significantly different between the groups, highlighting our robust statistical analyses. 

Our study has several limitations. First, our study is a cross-sectional, retrospective database that allows for the evaluation of associations and cannot determine causality. Clinical trials are needed to assess for any potential causal pathways. While there was a clinical feeding and fluid protocol (Supplemental Tables S1 and S2), clinicians could make feeding and fluid decisions that were off protocol based on the clinical status of the newborn. Though this is likely consistent with other clinical contexts, and, thus, increases the generalizability of our findings, it also may impact the findings related to enteral and parenteral fluids, further highlighting the need for clinical trials. While we have attempted to adjust for metrics of severity of illness, such as the need for respiratory support, birthweight, and gestational age, among other factors, the retrospective nature of this study design limits the ability to control for all potential confounders. Also, the clinical definitions for pulmonary hemorrhage included blood in the oropharynx, which may be due to alternative etiologies other than pulmonary hemorrhage. Additionally, we are unable to evaluate the macro- or micro-nutrient content of the parenteral fluids, and the common fluid support at SPHMMC is dextrose-containing fluids without lipids or amino acids. Therefore, it remains uncertain whether optimizing protein, fat, and/or carbohydrates or micro-nutrients (e.g., zinc, selenium, or other vitamins or minerals) may help offset or contribute to any risk incurred by parenteral fluids. 

## 5. Conclusions

Among moderately and very preterm newborns in Ethiopia, increasing first-week TFI is associated with adverse health outcomes, and this effect appears to be driven by parenteral intake rather than enteral intake. Notably, increasing first-week average enteral intake reduces the odds of developing adverse health outcomes, including a 70% reduction in mortality. Furthermore, differences in weight loss patterns from preterm newborns in the United States highlight that contextual differences, including the lack of humidified incubators and other resources, prevent the generalizability of findings from high-income settings to LMICs. Yet, LMICs have the highest burden of preterm birth and neonatal mortality. Due to the study design, our findings cannot be interpreted to depict a causal pathway but further add to the body of literature highlighting the need for robust clinical trials evaluating optimal early fluid and feeding management for vulnerable premature neonates in LMICs. Determining optimal enteral feeding volumes and advancement with or without parenteral fluids is of critical importance in improving health outcomes for preterm newborns in LMICs.

## Figures and Tables

**Table 1:** Maternal and infant factors related to maximal weight loss percentage categories.

Maternal or Infant Factor	≤13% Maximum Weight Loss	>13% Maximum Weight Loss	*p*-Value *
Total Number of Infants	328	162	-
Maternal Factors			
Maternal Age (years; median, IQR)	27 (5)	26 (7)	0.66
Prenatal Care: Yes	318 (97)	156 (98)	1.0
Antenatal Corticosteroids Provided: Yes	205 (62)	98 (62)	0.92
HIV: Yes	4 (1)	0 (0)	0.31
Syphilis: Yes	2 (1)	0 (0)	1.0
Cesarean Delivery: Yes	200 (62)	80 (49)	**0.01**
Number of Previous Pregnancies (median, IQR)	1 (2)	1 (2)	0.52
History of Preterm Birth: Yes	25 (8)	15 (9)	0.60
Urban: Yes	118 (36)	62 (38)	0.69
Chorioamnionitis: Yes	12 (4)	4 (2)	0.60
Pre-Eclampsia: Yes	136 (41)	51 (31)	**0.04**
Eclampsia: Yes	11 (3)	7 (4)	0.61
Oligohydramnios: Yes	12 (4)	2 (1)	0.16
Polyhydramnios: Yes	0 (0)	2 (1)	0.11
Gestational Diabetes: Yes	0 (0)	2 (1)	0.11
Hypertension: Yes	9 (3)	5 (3)	0.78
Tuberculosis: Yes	0 (0)	1 (1)	0.33
Hepatitis B: Yes	5 (2)	0 (0)	0.18
Infant Factors			
Gestational Age (weeks; median, IQR)	33 (2)	31 (2.8)	**<0.001**
Birthweight (g; median, IQR)	1465 (340)	1360 (371)	**<0.001**
5-min Apgar (median, IQR)	7 (1)	7 (1)	0.748
Sex: Female	150 (46)	74 (46)	1.00
Intrauterine Growth Restriction Diagnosis: Yes	51 (16)	10 (6)	**0.002**
Inborn: Yes	278 (85)	140 (86)	**0.69**
Singleton: Yes	234 (72)	88 (55)	**<0.001**
Received Respiratory Support Outside Delivery Room: Yes	314 (96)	159 (98)	0.20
7-day Average Total Fluid Intake Category			0.49
≤110 mL/kg birthweight/day	29 (9)	11 (7)	
>110 mL/kg birthweight/day	299 (91)	151 (93)	
7-day Average Total Fluid Intake (median, IQR)	129 (18)	136 (20)	**0.002**
Known Congenital Anomalies: Yes	5 (2)	2 (1)	1.0
Vitamin K administered: Yes	322 (98)	159 (98)	1.0

* Bolded *p*-values represent statistically significant values <0.05.

**Table 2:** Percent MWL > 13% vs. ≤13% and odds of developing adverse in-hospital outcomes.

	n/N (%)	Adjusted * OR	*p*-Value
In-hospital mortality ^#^			
MWL ≤ 13%	41/287 (14%)	1.00 (ref)	-
MWL > 13%	42/120 (35%)	1.30 (0.73, 2.32)	0.37
Suspected NEC ^#^			
MWL ≤ 13%	11/317 (3%)	1.00 (ref)	-
MWL > 13%	20/142 (14%)	2.99 (1.28, 6.95)	**0.01**
Culture-positive sepsis ^#^			
MWL ≤ 13%	68/260 (26%)	1.00 (ref)	-
MWL > 13%	61/101 (60%)	1.83 (1.14, 2.93)	**0.01**
ROP ^#^			
MWL ≤ 13%	61/267 (23%)	1.00 (ref)	-
MWL > 13%	63/99 (64%)	2.09 (1.28, 3.41)	**0.003**
Pulmonary hemorrhage ^#^			
MWL ≤ 13%	13/315 (4%)	1.00 (ref)	-
MWL > 13%	5/157 (3%)	0.42 (0.13, 1.42)	0.17
LOS **			
MWL ≤ 13%	Median 16	1.00 (ref)	-
MWL > 13%	Median 36	1.37 (1.24, 1.52)	**<0.001**

* Models included covariate adjustment for 7-day average total fluid intake, gestational age, birthweight, multiple birth (yes/no), mode of delivery of c-section (yes/no), pre-eclampsia (yes/no), respiratory support, and 5-min Apgar. ^#^ There were 20 missing values (3 missing c-section, 3 missing multiple birth, and 14 missing 5-min Apgar) for outcomes analyses for each of the following comorbidity assessments: in-hospital mortality, suspected NEC, culture-positive sepsis, ROP, and pulmonary hemorrhage. ** For length of stay, odds ratio is instead ratio of medians. LOS truncated at 60 days and log transformed; excluded if absconded or referred (n = 8). There were 28 cases of missing values for LOS analyses.

**Table 3:** 7-day average total fluid intake >110 mL/kg birthweight/day vs. <110 mL/kg birthweight/day and odds of developing adverse in-hospital outcomes.

	n/N (%)	Adjusted * OR	*p*-Value
In-hospital mortality ^#^			
≤110	1/39 (3%)	1.00 (ref)	-
>110	82/368 (22%)	3.75 (0.47, 29.61)	0.21
Suspected NEC ^#^			
≤110	0/40 (0%)	^∍^ (See Footnote)	
>110	31/419 (7%)		
Culture-positive sepsis ^#^			
≤110	6/34 (18%)	1.00 (ref)	-
>110	123/327 (38%)	2.11 (0.75, 6.94)	0.16
ROP ^#^			
≤110	2/38 (5%)	1.00 (ref)	-
>110	122/328 (37%)	4.70 (1.06, 20.89)	**0.04**
Pulmonary hemorrhage ^#,∍^			
≤110	0/40 (0%)	^∍^ (See Footnote)	
>110	18/432 (4%)		
LOS **			
≤110	9.5	1.00 (ref)	-
>110	21	1.39 (1.17, 1.65)	**<0.001**

* Models included covariate adjustment for MWL, gestational age, birthweight, multiple birth (yes/no), mode of delivery of c-section (yes/no), pre-eclampsia (yes/no), respiratory support, and 5-min Apgar. ^#^ There were 20 missing values (3 missing c-section, 3 missing multiple birth, and 14 missing 5-min Apgar) for outcomes analyses for each of the following comorbidity assessments: in-hospital mortality, suspected NEC, culture-positive sepsis, ROP, and pulmonary hemorrhage. ^∍^ There were no cases of suspected NEC or pulmonary hemorrhage in the >60 mL/kg birthweight/day group leading to perfect separation preventing evaluation of outcomes through regression modeling. ** For length of stay, odds ratio is instead ratio of medians. LOS truncated at 60 days and log transformed; excluded if absconded or referred (n = 8). There were 28 cases of missing values for LOS analyses. Bolded *p*-values represent statistically significant values < 0.05.

**Table 4:** The 7-day average enteral feeding or parenteral fluid intake of >60 mL/kg birthweight/day vs. ≤60 mL/kg birthweight/day and odds of developing adverse in-hospital outcomes.

Adverse In-Hospital Outcome	7-Day Average Enteral Intake			7-Day Average Parenteral Intake		
	n/N (%)	Adjusted * OR	*p*-Value	n/N (%)	Adjusted * OR	*p*-Value
**In-hospital mortality ^#^**						
≤60	76/260 (29%)	1.00 (ref)	-	4/151 (3%)	1.00 (ref)	-
>60	7/147 (5%)	0.32 (0.14, 0.78)	**0.01**	79/256 (31%)	5.32 (1.79, 15.86)	**0.003**
**Suspected NEC ^#^**						
≤60	30/306 (10%)	1.00 (ref)	-	1/154 (1%)	1.00 (ref)	-
>60	1/153 (1%)	0.12 (0.02, 0.91)	**0.04**	30/305 (10%)	6.91 (0.87, 54.96)	0.07
**Culture-positive sepsis ^#^**						
≤60	115/221 (52%)	1.00 (ref)	-	14/141 (10%)	1.00 (ref)	-
>60	14/140 (10%)	0.22 (0.11, 0.42)	**<0.001**	115/220 (52%)	4.37 (2.22, 8.60)	**<0.001**
**ROP ^#^**						
≤60	109/227 (48%)	1.00 (ref)	-	12/143 (8%)	1.00 (ref)	-
>60	15/139 (11%)	0.33 (0.17, 0.63)	**<0.001**	112/223 (50%)	3.82 (1.89, 7.72)	**<0.001**
**Pulmonary hemorrhage ^#,^** **^∍^**						
≤60	18/318 (6%)	^∍^ (see footnote)		0/155 (0%)	^∍^ (see footnote)	
>60	0/154 (0%)			18/317 (6%)		
LOS **						
≤60	26	1.00 (ref)	-	11	1.00 (ref)	-
>60	11	0.74 (0.66, 0.82)	**<0.001**	26	1.48 (1.33, 1.65)	**<0.001**

* Models are adjusted for residuals of enteral/parenteral fluids. Parenteral residuals are from PFA regressed on EFA and enteral residuals are from EFA regressed on PFA. All models also adjust for MWL, gestational age, birthweight, multiple birth (yes/no), mode of delivery of c-section (yes/no), pre-eclampsia (yes/no), respiratory support, and 5-min Apgar. ^#^ There were 20 missing values (3 missing c-section, 3 missing multiple birth, and 14 missing 5-min Apgar) for outcomes analyses for each of the following comorbidity assessments: in-hospital mortality, suspected NEC, culture-positive sepsis, ROP, and pulmonary hemorrhage. ^∍^ There were no cases of pulmonary hemorrhage in the >60 mL/kg birthweight/day group leading to perfect separation preventing evaluation of outcomes through regression modeling. ** For length of stay, odds ratio is instead ratio of medians. LOS truncated at 60 days and log transformed; excluded if absconded or referred (n = 8). There were 28 cases of missing values for LOS analyses. Bolded *p*-values represent statistically significant values < 0.05.

## Data Availability

The data presented in this study are available on request from the corresponding author due to data privacy restrictions.

## Supplementary Materials

The following supporting information can be downloaded at https://www.mdpi.com/article/10.3390/children12070872/s1, Table S1: Feeding volume for preterm neonatal feeding advancement; Table S2: Fluid volume for preterm neonatal fluid advancement. Figure S1: Flow diagram of participants meeting inclusion and exclusion criteria. Table S3: Maternal and infant factors related to those included versus excluded from data analysis. Table S4: Maximal weight loss among premature neonates at SPHMMC of differing gestational age categories. Table S5: Percent MWL < 5%, 5-≤13%, and >13%, and odds of developing adverse in-hospital outcomes. Figure S2: Graphical representation of weight loss trajectories evaluating median weight loss and corresponding 10th–90th percentile weight loss smoothed trajectories after birth stratified by gestational age. LOESS used for smoothing. Figure S3: Forest plots depicting adjusted odds ratios from multivariate regression modeling evaluating exposures on in-hospital adverse outcomes.

## Abbreviations

AGA	Appropriate for gestational age
BPD	Bronchopulmonary dysplasia
CI	Confidence interval
ELBW	Extremely low birthweight
EP	Extremely preterm
GA	Gestational age
GEE	Generalized estimating equations
ICH	Intracranial hemorrhage
IV	Intravenous
LOESS	Locally estimated scatterplot smoothing
NEC	Necrotizing enterocolitis
NICU	Neonatal intensive care unit
OR	Odds ratio
PDA	Patent ductus arteriosus
SGA	Small for gestational age
VLBW	Very low birthweight
