# Supplementary material for: Evaluation of First-Week Fluid Intake and Maximal Weight Loss Percentage with In-Hospital Adverse Outcomes Among Moderately and Very Preterm Newborns in Ethiopia
Source: Children (Basel). 2025 Jul 1;12(7):872. doi: 10.3390/children12070872 (PMC12293426; doi:10.3390/children12070872)
Supplement: Supplementary file 1 [file children-12-00872-s001.zip › children-3703554-supplementary.pdf]

Supplemental Materials

Supplemental Table S1: Feeding volume for preterm neonatal advancement of feeding volume			
Gestational age	Weight (grams)	Initial feeds*	Progression of feeds/advance as tolerated*
<28 weeks	<1000	10-20	10-20
28-<32 weeks	1000-<1500	20-30	20-30
32-<34 weeks	1500-1800	30	30
>34 weeks	>1800	30-60	35
*Of note, feeds and advancement are represented in mL/kg birthweight/day.			

Supplemental Table S2: Fluid volume for preterm neonatal fluid advancement			
Birthweight	Day 1*	Day 2*	Day 3-6*
<750 grams	100-140	120-160	140-200
750 - <1000 grams	100-120	120-140	130-180
1000 - <1,500 grams	80-100	100-120	120-160
*Of note, fluids are represented in mL/kg birthweight/day.			

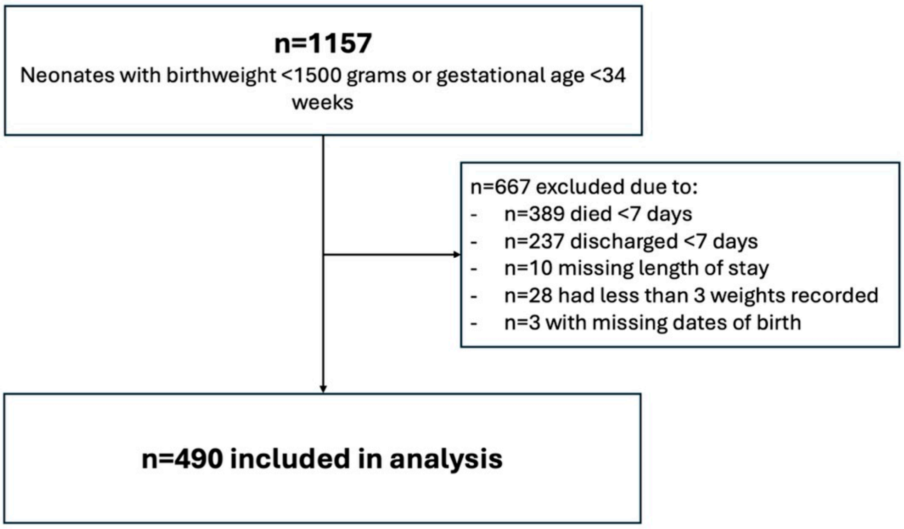

Supplemental Figure S1: Flow diagram of participants meeting inclusion and exclusion criteria.

Supplemental Table S3: Maternal and infant factors related to those included versus excluded from the data analysis

Maternal or Infant Factor	Excluded	Included	p-value*
Total Number of Infants	667	490	-
**Maternal Factors**			
Maternal Age (years; median, IQR)	26 (7)	27 (6)	0.39
Prenatal Care: Yes	637 (96)	474 (97)	0.42
Antenatal Corticosteroids Provided: Yes	401 (61)	303 (62)	0.58
HIV: Yes	12 (2)	4 (1)	0.21
Syphilis: Yes	2 (0)	2 (0)	1
Cesarean Delivery: Yes	362 (55)	280 (57)	0.34
Number of Previous Pregnancies (median, IQR)	1 (2)	1 (2)	0.76
History of Preterm Birth: Yes	45 (7)	40 (8)	0.43
Urban: Yes	245 (37)	180 (37)	1
Chorioamnionitis: Yes	11 (2)	16 (3)	0.08
Pre-Eclampsia: Yes	253 (38)	187 (38)	0.95
Eclampsia: Yes	24 (4)	18 (4)	1
Oligohydramnios: Yes	15 (2)	14 (3)	0.57
Polyhydramnios: Yes	2 (0)	2 (0)	1
Gestational Diabetes: Yes	9 (1)	2 (0)	0.13
Hypertension: Yes	18 (3)	14 (3)	0.86
Tuberculosis: Yes	1 (0)	1 (0)	1
Hepatitis B: Yes	3 (0)	5 (1)	0.30
**Infant Factors**			
Gestational Age (weeks; median, IQR)	31 (3)	32 (2)	**<0.01**
Birthweight (g; median, IQR)	1370 (642.5)	1448 (505)	0.09
5-minute Apgar (median, IQR)	7 (1)	7 (1)	**0.049**
Sex: Female	302 (45)	224 (46)	0.95
Intrauterine Growth Restriction Diagnosis: Yes	50 (8)	61 (13)	**<0.01**
Inborn: Yes	561 (84)	418 (85)	0.74
Singleton: Yes	477 (72)	322 (66)	**0.04**
Received Respiratory Support Outside Delivery Room: Yes	594 (89)	473 (97)	**<0.01**
Known Congenital Anomalies: Yes	22 (3)	7 (1)	0.06
Vitamin K administered: Yes	654 (98)	481 (98)	0.82

Supplemental Table S4. Maximal Weight Loss Among Premature Neonates at SPHMMC of Differing Gestational Age Categories					
**Gestational Age Categories**	**<30 weeks**	**30-31 weeks**	**32-33 weeks**	**≥34 weeks, <1500g**	**Total**
**Number of babies**	50	143	240	57	490
**Mean MWL %**	15.4	13	9.6	6.2	10.8
**Median MWL % (IQR)**	14.7 (11.5, 20)	12.4 (8.9, 16)	9.3 (5.6, 12.9)	5.4 (2.7, 8.9)	10.4 (6.1, 14.7)
**Median (IQR) day of MWL -days since birth**	5 (4, 6.8)	6 (4, 7)	6 (4, 7)	4 (2, 5)	5.5 (4, 7)

**Supplemental Table S5: Percent MWL <5%, 5-≤13% and >13% and odds of developing adverse in-hospital outcomes**			
	n/N (%)	Adjusted* OR	p-value
In-hospital mortality[#]			
MWL <5%	15/77 (19%)	1.99 (0.92, 4.31)	0.08
MWL 5-≤13%	26/210 (12%)	1.00 (ref)	-
MWL >13%	42/120 (35%)	1.56 (0.84,2.92)	0.16
Suspected NEC[#]			
MWL <5%	3/89 (3%)	0.85 (0.21,3.43)	0.82
MWL 5-≤13%	8/228 (4%)	1.00 (ref)	-
MWL >13%	20/142 (14%)	2.86 (1.14,7.15)	**0.02**
Culture positive sepsis[#]			
MWL <5%	14/78 (18%)	0.54 (0.27,1.09)	0.09
MWL 5-≤13%	54/182 (30%)	1.00 (ref)	-
MWL >13%	61/101 (60%)	1.60 (0.98,2.62)	0.058
ROP[#]			
MWL <5%	17/75 (23%)	0.86 (0.44,1.69)	0.66
MWL 5-≤13%	44/192 (23%)	1.00 (ref)	-
MWL >13%	63/99 (64%)	2.02 (1.21,3.37)	**<0.01**
Pulmonary hemorrhage[#]			
MWL <5%	5/87 (6%)	1.11 (0.28,4.35)	0.88
MWL 5-≤13%	8/228 (4%)	1.00 (ref)	-
MWL >13%	5/157 (3%)	0.44 (0.12,1.55)	0.20
LOS**[&]			
MWL <5%	Median 15	1.00 (0.88,1.13)	0.98
MWL 5-≤13%	Median 16	1.00 (ref)	-
MWL >13%	Median 36	1.37 (1.23,1.53)	**<0.01**

*Models included covariate adjustment for 7-day average total fluid intake, gestational age, birthweight, multiple birth (yes/no), mode of delivery c-section (yes/no), pre-eclampsia (yes/no), respiratory support, 5-minute Apgar.

[#]There were 22 missing values for outcomes analyses for each of the following comorbidity assessments: in-hospital mortality, suspected NEC, culture-positive sepsis, ROP, and pulmonary hemorrhage.

**For length of stay, the odds ratio is instead the ratio of medians. LOS truncated at 60 days and log transformed; excluded if absconded or referred (n=8). There were 29 cases of missing values for the LOS analyses.

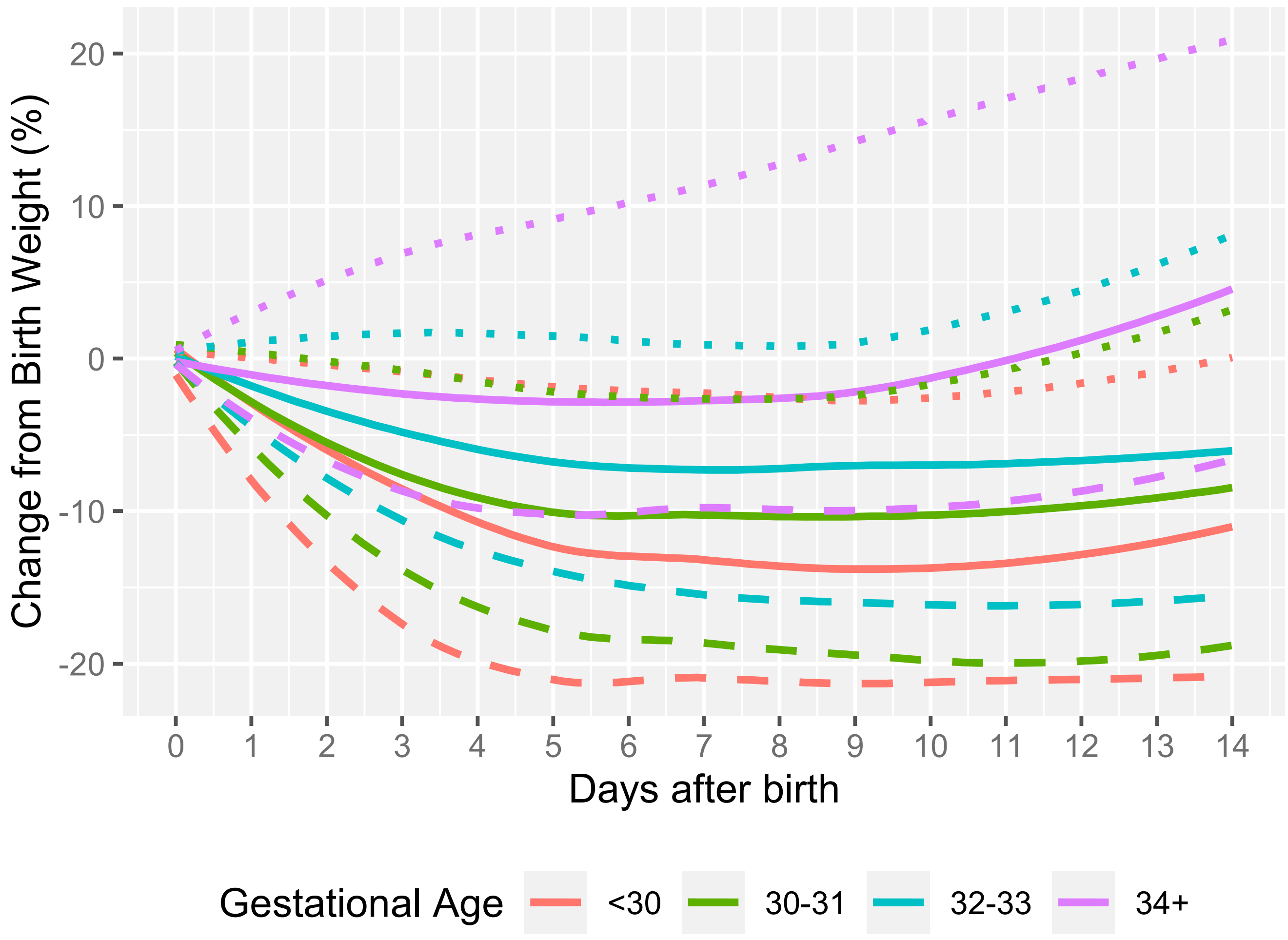

Supplemental Figure S2. Graphical representation of the weight loss trajectories evaluating the median weight loss and corresponding 10th-90th percentile weight loss smoothed trajectories after birth stratified by gestational age. LOESS used for smoothing.

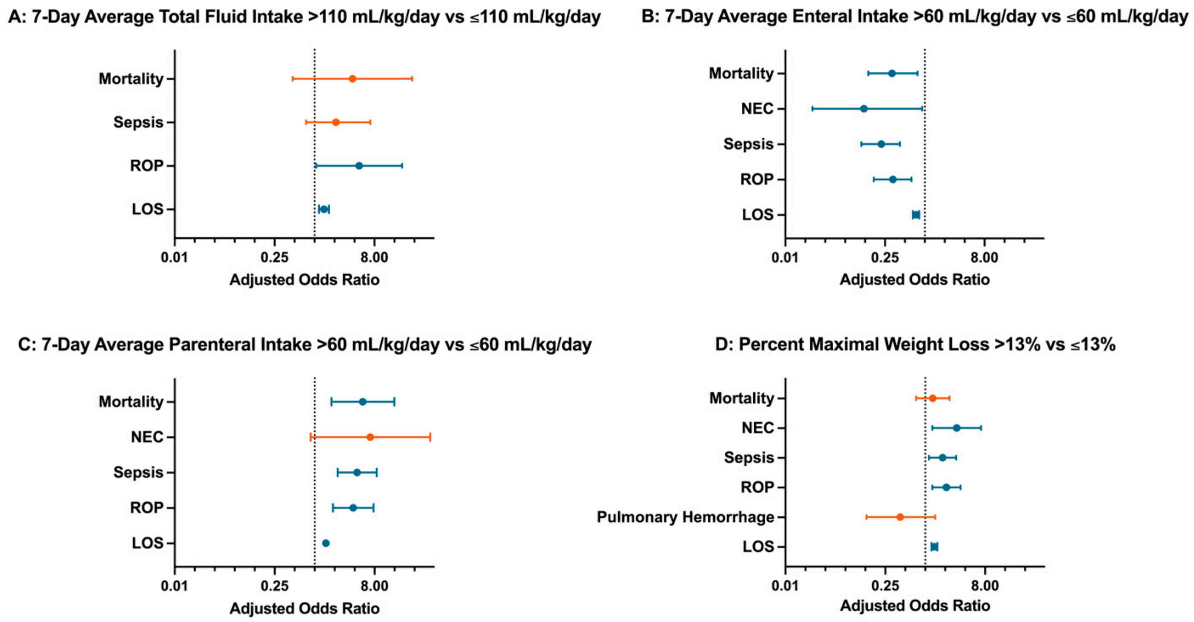

Supplemental Figure S3: Forest plots depicting the adjusted odds ratios from multivariate regression modeling evaluating exposures on in-hospital adverse outcomes. Exposures were as follow: (a) 7-day average total fluid intake evaluating >110 mL/kg birthweight/day vs ≤110 mL/kg birthweight/day, (b) 7-day average enteral feeding intake evaluating >60 mL/kg birthweight/day vs ≤60 mL/kg birthweight/day, (c) 7-day average parenteral fluid intake evaluating >60 mL/kg birthweight/day vs ≤60 mL/kg birthweight day, and (d) percent maximal weight loss evaluating >13% MWL vs ≤13% MWL. Models for percent MWL and TFI included covariate adjustment for TFI (for MWL) and MWL (for TFI). Models for enteral feeds and parenteral fluids included: Enteral residuals are from 7-day average enteral feeding intake regressed on 7-day average parenteral fluid intake and parenteral residuals are from the 7-day average parenteral fluid intake regressed on 7-day average enteral feeding intake. All 4 models also adjust for MWL, gestational age, birthweight, multiple birth (yes/no), mode of delivery, and pre-eclampsia (all variables that had a p-value of <0.10 at baseline between groups). For length of stay, the odds ratio is instead the ratio of the medians. LOS truncated at 60 days and log transformed; excluded if absconded or referred. Due to 0 instances of necrotizing enterocolitis in those with TFI ≤110 mL/kg/day, no aOR or p-value could be calculated. Due to all cases of pulmonary hemorrhage occurring in neonates with those with a TFI >110 mL/kg/day, parenteral fluids >60 mL/kg/day and enteral feeds ≤60 mL/kg/day, no aOR or p-value could be calculated for these analyses and respective Forest plots. **Significant findings are depicted in teal.** NEC: Necrotizing enterocolitis; ROP: Retinopathy of Prematurity; LOS: Length of stay
